# The REACH VET Program and Mortality Outcomes Among Veterans at High Risk of Suicide

**DOI:** 10.1001/jamanetworkopen.2025.19513

**Published:** 2025-07-08

**Authors:** Kallisse R. Dent, Samantha Cooper, John F. McCarthy

**Affiliations:** 1 Serious Mental Illness Treatment Resource and Evaluation Center, Office of Mental Health, Department of Veterans Affairs, Ann Arbor, Michigan; 2 Office of Suicide Prevention, Department of Veterans Affairs, Ann Arbor, Michigan; 3 Department of Psychology, University of Michigan, Ann Arbor, Michigan; 4 Department of Psychiatry, University of Michigan, Ann Arbor, Michigan

## Abstract

This cohort study investigates the association of the Veterans Health Administration Recovery Engagement and Coordination for Health–Veterans Enhanced Treatment (REACH VET) program with suicide and mortality outcomes among veterans at high risk for suicide.

## Introduction

To support veterans at high risk for suicide,^1^ the Veterans Health Administration (VHA) developed a suicide risk prediction algorithm^2^ and implemented the Recovery Engagement and Coordination for Health–Veterans Enhanced Treatment (REACH VET) program.^3^ It identifies VHA patients at high risk for suicide (top 0.1% risk tier), providing outreach and care coordination. An effectiveness evaluation of the REACH VET program documented reductions in nonfatal suicide attempts but did not identify associations with suicide mortality.^4^ Those mortality analyses were limited to a 6-month period for a subset of the study cohort. This study evaluates the association of REACH VET with mortality outcomes through 24 months for an expanded cohort.

## Methods

Analyses were conducted as a part of Veterans Affairs Office of Suicide Prevention operations, and institutional review board review was not required. We followed the STROBE reporting guideline for cohort studies.

A differences-in-differences design was used to evaluate the association of REACH VET with suicide, external-cause mortality, and all-cause mortality. The intervention group included patients who were identified in the top 0.1% risk tier during the REACH VET implementation period (March 2017 to June 2021). A subthreshold high–risk tier group (top 0.3%-0.1% risk tier) was identified as a control group. Comparable groups were also identified during a preintervention period (March 2014 to December 2015). Mortality was assessed using national death certificate data through 2021 per the Veterans Affairs/Department of Defense Mortality Data Repository.

Cox proportional hazards regression was used to estimate the association of the REACH VET program with mortality outcomes across 6-, 12-, and 24-month follow-up periods adjusting for age and sex. The eMethods in Supplement 1 provides detailed information on study methods. Significance was assessed using 2-tailed χ^2^ tests, with α = .05.

## Results

The analytic cohort consisted of 266 246 observations (92.3% male; 0.6% American Indian or Alaskan Native, 0.9% Asian, Native Hawaiian, or Other Pacific Islander, 14.2% Black, 81.4% White, 1.6% multiple racial groups, and 1.3% unknown or missing; 91.0% non-Hispanic; mean [SD] age, 51.2 [15.0] years). Accounting for potential period and cohort differences, inclusion in the REACH VET program was not associated with suicide mortality (6-month hazards ratio [HR], 1.18 [95% CI, 0.83-1.69]; 12-month HR, 1.25 [95% CI, 0.94-1.67]; 24-month HR: 0.92 [95% CI, 0.68-1.24]) (Table). Similarly, inclusion in REACH VET was not associated with risk for external-cause mortality (6-month HR, 0.99 [95% CI, 0.80-1.23]; 12-month HR, 1.07 [95% CI, 0.91-1.27]; 24-month HR, 1.06 [95% CI, 0.90-1.25]) or all-cause mortality (6-month HR, 1.03 [95% CI, 0.93-1.15]; 12-month HR, 1.04 [95% CI, 0.95-1.13]; 24-month HR, 1.03 [95% CI, 0.95-1.12]).

**Table.** Association of the REACH VET Program With Mortality Outcomes

Outcome	Observations, No.		Mortality, HR (95% CI)		
	Total	With outcome	Pre–REACH VET Era^a^	Post–REACH VET Era^a^	DiD^b^
Suicide					
6 mo	266 246	566	1.40 (1.04-1.87)	1.65 (1.34-2.02)	1.18 (0.83-1.69)
12 mo	247 481	841	1.34 (1.06-1.69)	1.68 (1.42-2.00)	1.25 (0.94-1.67)
24 mo	178 048	904	1.58 (1.22-2.05)	1.46 (1.25-1.70)	0.92 (0.68-1.24)
External-cause mortality					
6 mo	266 246	1603	1.34 (1.11-1.60)	1.33 (1.18-1.49)	0.99 (0.80-1.23)
12 mo	247 481	2533	1.26 (1.10-1.45)	1.35 (1.23-1.49)	1.07 (0.91-1.27)
24 mo	178 048	3130	1.31 (1.14-1.51)	1.39 (1.28-1.50)	1.06 (0.90-1.25)
All-cause mortality					
6 mo	266 246	6976	0.80 (0.73-0.87)	0.82 (0.78-0.87)	1.03 (0.93-1.15)
12 mo	247 481	11 081	0.85 (0.79-0.91)	0.88 (0.84-0.92)	1.04 (0.95-1.13)
24 mo	178 048	13 686	0.91 (0.84-0.97)	0.93 (0.90-0.97)	1.03 (0.95-1.12)

Abbreviations: DiD, difference in difference; HR, hazard ratio; REACH VET, Recovery Engagement and Coordination for Health–Veterans Enhanced Treatment.

^a^ The association with mortality, adjusted for age and sex, is given for the top 0.1% risk tier vs the top 0.3% to 0.1% risk tier within the period prior to (pre–REACH VET era) or during (post–REACH VET era) REACH VET implementation.

^b^ The DiD association of the REACH VET program with mortality is given, adjusted for age and sex, with the HR equivalent to the ratio of the post–REACH VET era HR to the pre–REACH VET era HR.

## Discussion

Consistent with previous reports,^4^ this cohort study did not observe associations of REACH VET program inclusion with subsequent mortality outcomes. Findings highlight the complexities of suicide prevention. Although the REACH VET program has been associated with improvements in suicide-related risk factors, including suicide attempts,^4^ this study replicated prior observations that program inclusion was not associated with mortality outcomes. Differential associations of the REACH VET program with nonfatal and fatal suicide attempts may be influenced by differences in characteristics of patients who attempt suicide vs those who die from suicide. Compared with patients with nonfatal suicide attempts, individuals who die by suicide tend to use more lethal methods and be older, more often male, and have more medical morbidity.^5^ Considered alongside our findings, this suggests that the REACH VET program may be less effective for specific subpopulations of VHA patients at high risk, such as males or those using more lethal methods. Additional research is needed to evaluate these hypotheses.

A limitation of this study was its low power to detect small associations for rare outcomes; power analyses suggest we would need approximately 1.4 million patients to observe a 10% difference in suicide risk across a 12-month follow-up. Furthermore, analyses may not have fully accounted for underlying group differences in mortality risks across time. Although REACH VET care coordination and outreach are important for improving mental health outcomes and preventing suicide attempts, additional approaches are needed to enhance suicide prevention for patients at high risk.

## Notes

Supplement 1. **eMethods.** Methodological Details for the Assessment of the REACH VET Program and Mortality Outcomes
eReferences.

Supplement 2.
Data Sharing Statement
