# Supplementary material for: The REACH VET Program and Mortality Outcomes Among Veterans at High Risk of Suicide
Source: JAMA Netw Open. 2025 Jul 8;8(7):e2519513. doi: 10.1001/jamanetworkopen.2025.19513 (PMC12238888; doi:10.1001/jamanetworkopen.2025.19513)
Supplement: Supplement 1. — eMethods. Methodological Details for the Assessment of the REACH VET Program and Mortality Outcomes eReferences. [file jamanetwopen-e2519513-s001.pdf]

# Supplemental Online Content

Dent KR, Cooper S, McCarthy JF. The REACH VET program and mortality outcomes among veterans at high risk of suicide. *JAMA Netw Open*. 2025;8(7):e2519513. doi:10.1001/jamanetworkopen.2025.19513

**eMethods.** Methodological Details for the Assessment of the REACH VET Program and Mortality Outcomes

**eReferences.**

This supplemental material has been provided by the authors to give readers additional information about their work.

© 2025 Dent KR et al. *JAMA Network Open*.

**eMethods.** Methodological Details for the Assessment of the REACH VET Program and Mortality Outcomes

Cohort. A differences-in-differences approach was used to test whether the REACH VET program had significant associations with mortality outcomes. To implement a differences-in-differences approach, we utilized the suicide risk prediction algorithm developed by the Veterans Health Administration to identify those meeting eligibility criteria for the program (top 0.1% of suicide risk) during the implementation period (March 2017-June 2021) and in a period prior to implementation (March 2014-December 2015). We also identified individuals in a subthreshold control group (top 0.3-0.1% of suicide risk tier) across the same periods. Within each time period, an individual's first instance in the top 0.1% and subthreshold groups were identified. Individuals could contribute to both the top 0.1% and subthreshold groups if they were identified for the subthreshold group prior to being identified as in the top 0.1% group. They could also contribute to both the pre-REACH VET implementation period and the post-REACH VET implementation period.

Outcome. Mortality was assessed using death certificate data (from the National Death Index) through 2021 compiled in the Veteran Affairs/ Department of Defense Mortality Data Repository. ICD-10 cause of death codes used to identify suicide included X60-X84, Y87.0, U03. Those used to identify external-causes of death included V01-Y36, Y85-Y87, Y89, U01-U03.

Study Covariates. Age and sex information was derived from VA administrative data sources. VHA patients with missing data on study variables were excluded. To provide additional context about the patients included in our study, VA administrative data sources were also used to derive information on race (American Indian/Alaskan Native, Asian/Native Hawaiian/Pacific Islander, Black, Multiple Racial groups, White, Unknown/missing), and ethnicity (Hispanic, Non-Hispanic, Unknown/missing), however, these variables were not included in statistical modeling.

Risk time. VHA patients identified during the pre-REACH VET implementation period started contributing risk time when they entered the cohort, while those in the post-REACH VET implementation period started contributing risk time when their data was released to the field. The data release date was used in the implementation period since this corresponds to the day that clinicians could start providing outreach and care-coordination to patients identified by the algorithm. A comparable date was not available in the pre-implementation period. Risk-time ended on the individual's date of death or the end of the follow-up period, whichever came first. Those in the subthreshold risk tier were also censored if they were later identified in the top 0.1% risk tier.

Statistical analysis. Cox proportional hazards regression was used to estimate the association of the REACH VET program with mortality outcomes. Indicators for the observation's time period (pre- or post-REACH VET implementation) and risk group (top 0.1% or subthreshold) were created. An interaction term between time period and risk group was used to test the association of the REACH VET program with mortality outcomes. All models accounted for age and sex. The robust sandwich estimator was used to account for individuals who appeared in the model multiple times (different risk tier and/or time periods).

Parallel trend assumption. Results derived from a differences-in-differences study design rely on the parallel trend assumption. Within this study, the parallel trend assumption required that trends in mortality for the top 0.1% and subthreshold groups were parallel (i.e., increase or decrease at the same rate) prior to the implementation period (**Figure 1**). This was tested using data from the pre-implementation era with a 6-month follow-up period. An interaction term between an indicator for the top 0.1% risk tier and year was used to test parallel trends. We found that there was no significant difference

© 2025 Dent KR et al. *JAMA Network Open*.

in the trend in 6-month suicide risk (March 2015-December 2015) across the top 0.1% and subthreshold groups in the pre-implementation era (p-value=0.40). There were also no significant non-parallel trends for external causes of death (p-value=0.50) or all-cause mortality (p-value=0.09).

While our tests of the parallel trend assumption suggest that there are parallel mortality trends across the top 0.1% risk group and subthreshold group during the pre-implementation period (2014-2015), it is plausible that these trends did not hold during the implementation period, 2017-2021. During this period suicide rates among VHA patients generally increased.[1] If risk changed at a greater rate for those in the high-risk tier (top 0.1%) when compared to the subthreshold risk tier (top 0.3%), then this may have masked significant associations of the REACH VET program with mortality.

Power. In response to reviewer feedback, post-hoc power analysis for survival data was conducted. We identified the number of observations required to identify a 10% difference in suicide risk among those receiving REACH VET, assessing suicide mortality across a 12-month period with 80% power. Power analyses suggested that we would need to identify approximately 4,839 deaths by suicide during the follow-up period. If suicide occurred at a rate similar to our present study, we would need approximately 1.4 million observations in our analytic cohort to observe a 10% difference in suicide risk.

© 2025 Dent KR et al. *JAMA Network Open*.

**eReferences.**

Office of Suicide Prevention, Department of Veteran Affairs. 2024 National Veteran Suicide Prevention Annual Report Part 2 or 2: Report Findings. Accessed December 27, 2024. https://www.mentalhealth.va.gov/docs/data-sheets/2024/2024-Annual-Report-Part-2-of-2_508.pdf
