# Supplementary material for: The REACH VET Program and Mortality Outcomes Among Veterans at High Risk of Suicide
Source: JAMA Netw Open. 2025 Jul 8;8(7):e2519513. doi: 10.1001/jamanetworkopen.2025.19513 (PMC12238888; doi:10.1001/jamanetworkopen.2025.19513)
Supplement: Supplement 2. — Data Sharing Statement [file jamanetwopen-e2519513-s002.pdf]

Data Sharing Statement

Dent. The REACH VET Program and Mortality Outcomes Among Veterans at High Risk of Suicide. *JAMA Netw Open*. Published online July 8, 2025. doi:10.1001/jamanetworkopen.2025.19513

Data
Data available: No

Additional Information
Explanation for why data not available: Data used for this study include personal health information and are not available for public use.
